# Preliminary Classification of Selected Farmland Habitats in Ireland Using Deep Neural Networks

**DOI:** 10.3390/s22062190

**Published:** 2022-03-11

**Authors:** Lizy Abraham, Steven Davy, Muhammad Zawish, Rahul Mhapsekar, John A. Finn, Patrick Moran

**Affiliations:** 1 Walton Institute for Information and Communication Systems Science, Waterford Institute of Technology, X91 WR86 Waterford, Ireland; steven.davy@waltoninstitute.ie (S.D.); muhammad.zawish@waltoninstitute.ie (M.Z.); rahul.mhapsekar@waltoninstitute.ie (R.M.); 2 Teagasc, Environment Research Centre, Johnstown Castle, Y35 TC97 Wexford, Ireland; john.finn@teagasc.ie; 3 Forest Environmental Research and Services Ltd. (FERS), Kilberry, C15 R6Y3 Navan, Ireland; pat.moran@fers.ie

**Keywords:** farmland habitat, habitat classification, drone images, base-learner, meta-learner, stacked ensemble model

## Abstract

Ireland has a wide variety of farmlands that includes arable fields, grassland, hedgerows, streams, lakes, rivers, and native woodlands. Traditional methods of habitat identification rely on field surveys, which are resource intensive, therefore there is a strong need for digital methods to improve the speed and efficiency of identification and differentiation of farmland habitats. This is challenging because of the large number of subcategories having nearly indistinguishable features within the habitat classes. Heterogeneity among sites within the same habitat class is another problem. Therefore, this research work presents a preliminary technique for accurate farmland classification using stacked ensemble deep convolutional neural networks (DNNs). The proposed approach has been validated on a high-resolution dataset collected using drones. The image samples were manually labelled by the experts in the area before providing them to the DNNs for training purposes. Three pre-trained DNNs customized using the transfer learning approach are used as the base learners. The predicted features derived from the base learners were then used to train a DNN based meta-learner to achieve high classification rates. We analyse the obtained results in terms of convergence rate, confusion matrices, and ROC curves. This is a preliminary work and further research is needed to establish a standard technique.

## 1. Introduction

Habitat mapping can be utilized in a variety of applications in nature conservation. They serve as a guiding principle for monitoring inventories of natural areas, curating the networks of protected areas, environmental impact assessment, management planning, and target setting for ecological restoration. However, most such applications still rely on field-based methods. The research in this area is increasingly focused on the data available from satellite imagery. The main works are related to forest and vegetation mapping using LANDSAT images, WorldView-2, Sentinel-2, IKONOS, GeoEye, MERIS, radar, and LiDAR images [1,2,3,4,5,6,7]. Earth observation data offers new opportunities for environmental sciences and is transforming artificial intelligence-based methodologies because of the massive data with spatial, spectral, and temporal variations available from satellite sensors [8,9,10]. The use of traditional remote sensing images for land use monitoring and mapping has been widely used in many types of research from the early 2000s onwards [11,12,13]. The works of Cheng et al. [14] and Xie et al. [1] describe state-of-the-art technologies and possible datasets for land cover mapping and classification. There is increasing demand for applications to monitor ecosystems and assess their seasonal variations in the twenty-first century as climate change and global warming are making significant impacts all over the world [15,16]. To implement effective biodiversity conservation and climate protection practices on an international and national scale, accurate mapping of habitats plays a vital role. One of the prominent works in this direction is the European Union’s (EU) Copernicus Programme [17]. Through this program, global data can be obtained in real-time using airborne, ground-based, and seaborne-based systems, which can also be used for local and regional needs efficiently.

Many works have been published using the Copernicus Land Monitoring Service (CLMS), due to it being free and openly accessible to users [18,19,20]. The main constraint is that, since the spatial resolution of CLMS is at EU-level, it has limitations for more differentiated habitat identification at the scale of individual member countries. Another distinguished EU programme is the European Nature Information System (EUNIS) which accumulates the European data from multiple databases and is extensively used as the main reference for the research in ecology and conservation [21,22]. While the EU Copernicus Programme supports research mainly in six thematic areas, namely land, marine, atmosphere, climate change, emergency management, and security, EUNIS has a dedicated directive for habitat classification. Indeed, it is the main comprehensive European hierarchical classification of habitats that covers not only marine but also terrestrial realms from early 1990 onwards [23,24,25,26]. It has become one of the key elements of the INSPIRE (Infrastructure for Spatial Information in Europe) Directive [27] which aims to create an EU spatial data infrastructure for policies or approaches that might affect the environmental structure. It is also the main contributor to Resolution 4 (1996) of the Bern Convention on endangered natural habitat types released in 1996 which was subsequently revised in 2010 and 2014 [28]. It also assists the Natura 2000 process (EU Birds and Habitats Directives), the development of indicators in the European Economic Area (EEA) core set, and the environmental reporting connected to EEA reporting activities [29]. Later, for establishing a solid database on a continental scale, it has been renewed and thus used in a major scale for studying land cover usage, vegetation, forest and habitat mapping [30,31,32].

However, the scarcity of publicly available datasets with remote sensing images at an appropriate scale and resolution has hindered the development of new models and methods using deep learning techniques as they demand massive heterogeneous data. Rapid advancements in unmanned aerial vehicle (UAV) technology have allowed highly accurate data collection with a wide variety of sensors. Many recent works in this area are focused on UAV images acquired using RGB, multispectral, hyperspectral, and thermal imaging cameras [33,34,35,36,37]. The works are not only contributing new methodologies but also high-resolution image datasets to the public domain, which in turn support applications that require in-depth analysis. The advantages of UAV imagery include ultra-high spatial resolution, low altitude images allowing detection of fine details, flexibility in using diverse sensors that can acquire different ranges of the spectrum, and ease of collection compared to data collected by fieldwork which is often limited by the logistical effort of field surveys. Images from UAVs are routinely used for monitoring diseases, crop nutrition, forest fire, hydrology, and topography analysis for drainage and road construction, creating high-resolution vegetation, forest, and habitat maps, 3-D mapping, and estimation of tree height and surveys over inaccessible areas. Rather than automated methods, these applications are still mostly dependent on fieldworks.

For all the use case scenarios, studies are concentrated on the forest, vegetation, land cover and habitat mapping; also for observing climate changes, identifying crop and soil conditions, water content determination, and drought monitoring. Extensive research is still required in automated habitat mapping methods that deal with the use of drones in farm-scale image collection and the use of deep learning techniques for the identification of habitats. Habitat mapping is always a challenging task; habitats frequently merge or grade from one to another, or form complex mosaics, with the result that a continuum of variation often exists within and between different habitat types which blur the distinctions between habitats. Although it reflects some natural phenomenon, however, in some cases, disturbance and damage blur the contrast among habitats. Another complicating factor is that typical farmland habitats have a dynamic nature due to their varying attributes across different seasons of the year e.g., hay meadows, reedbeds, areas of dense bracken, or turloughs. Fertilizer use and heavy grazing could also be the reason for a significant alteration in habitat structure, function, quality, and species composition. Therefore, even the complex deep learning algorithms fail to distinguish between different habitat types. The lack of availability of image datasets in the public domain is another problem. Yasir et al. [38] describe available datasets that can be used for various habitat mapping problems but it doesn’t contain any farmland datasets. Some of the recent works in this regard are habitat mapping of marine, land, and benthic zones [39,40,41,42,43,44,45,46,47,48,49].

In this work, we tried to classify Irish farmlands using self-collected drone images. Farming in most of Ireland ranges from highly managed arable land in the east to smaller wetter fields in the west [50,51] and farmland habitats are an integral part of Irish biodiversity [52,53]. However, there is nearly no authentic work or datasets available for the automated classification of Irish farmlands in the public domain.

The specific objectives of this study were (i) to collect and label habitat images of representative Irish farmlands collected using UAVs, (ii) to develop a machine learning method for the digital classification of farmland habitat types, and (iii) assess the effectiveness of the developed classification scheme for habitat identification in the Irish farmlands. The paper is organized as follows: Section 2 describes the developed habitat classification method systematically. The method is validated in Section 3 using various experiments conducted on the dataset and comparing those with existing works. Analysis of the results is presented in Section 4. Finally, the work is concluded in Section 5.

## 2. Materials and Methods

**Figure 1:**
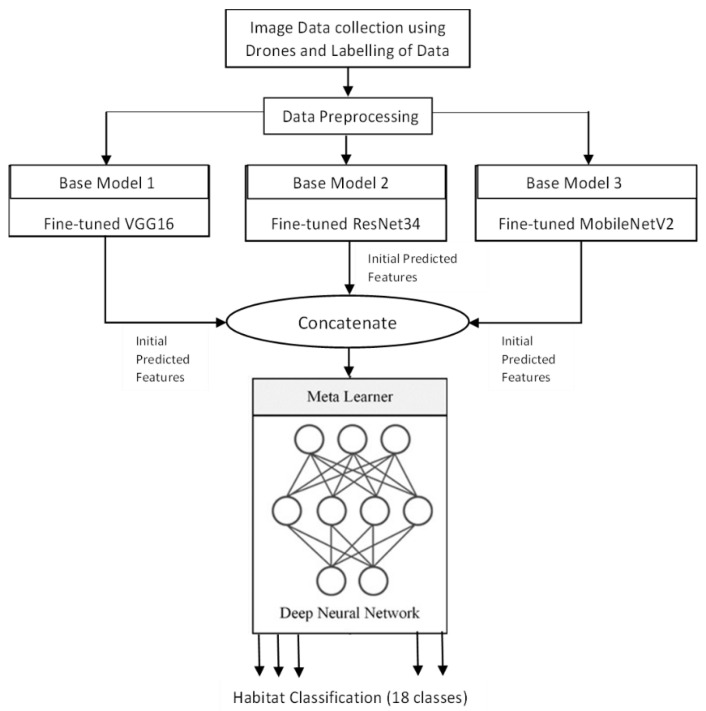
Workflow of the System.

The overall workflow of the proposed approach is presented in Figure 1. The workflow is explained in detail in the below subsections.

### 2.1. Data Collection

The task comprised collecting images of a variety of selected habitats [48] in Ireland from a drone using a high-resolution imaging camera. For this purpose, a DJI Mavic 2 Zoom drone was used. It has a CMOS imaging sensor of 1/2.3-inch, 12-megapixels resolution with up to four-times zoom, including a two-times optical zoom (24–48 mm) which makes it suitable for aerial photography. The field of view (FOV) is about 48° to 83°. The lens is auto-focus with a focal length of approximately 4×. The aspect ratio was kept at 3:2 for capturing the images. Different fields within the farm have been identified, and the drone collected a low number (approximately fewer than 10) of replicate images from that field, before moving on to the next field within the farm. Thus, the method sampled different fields with different habitat types within the farm. For this work, the images were captured from July to September 2021 at around noon. The height of the flight was set at approximately 20 m above ground level.

**Figure 2:**
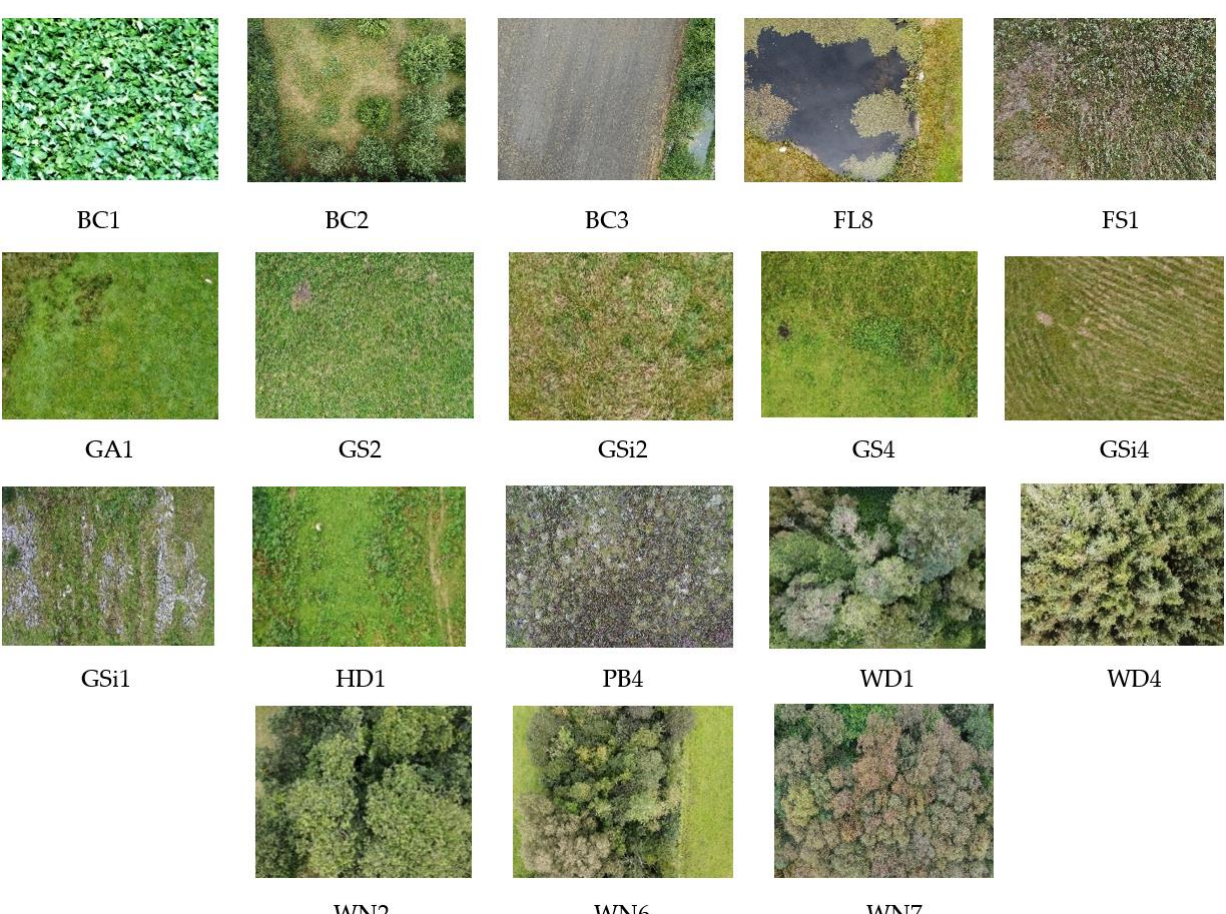
Sample images of 18 different habitats under study.

A total of 2233 images were collected ranging across 18 different classes. Habitat categories that belong to these 18 classes can be broadly grouped into 6 different types [47,48]. Table 1 describes the habitat types among 18 different classes covered by the image dataset. Figure 2 provides one sample image from each of the habitats along with the habitat type of the study area.

**Figure 3:**
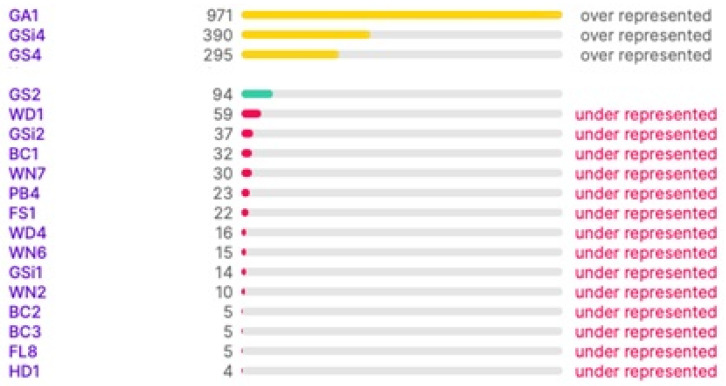
Class Balance between Habitat Types.

The samples were labelled by JAF and PM [52]. The master information sources that have been referenced for labelling the images are the guides published by the Irish Heritage Council [52,54]. The manuals provide a standard scheme and protocol for identifying, describing, and classifying wildlife habitats in Ireland. Figure 3 illustrates the range of class types and the number of images in each type in the dataset after labelling. It is noted that the distribution of the images to class types is not ideal for training; however, this can be improved upon in the future by collecting more images. In this work, this is dealt with the task by boosting the data in the under-represented classes.

### 2.2. Data Cleaning and Preprocessing

**Figure 4:**
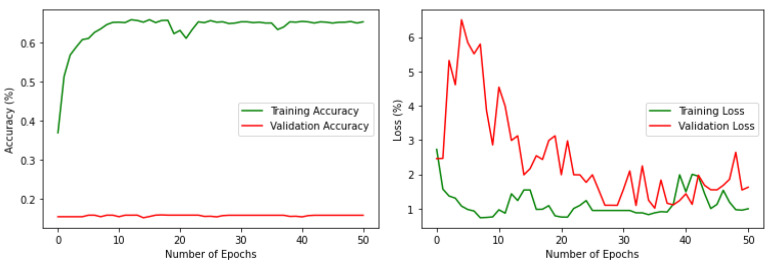
Accuracy and loss, respectively, for the imbalenced data.

Some datasets contain class imbalance and have far more instances than others (Figure 3). Achieving high classification accuracy with this data was difficult. However, to assess the performance with this imbalanced data, a simple classification was performed with a VGG16 pre-trained model by changing the number of nodes in the final classification layer to 18. 20% of the data has been used for validation. The results are shown in Figure 4. It can be noted that the performance is poor in terms of both accuracy and loss. The validation accuracy is less than 20%, which clearly outlines that if the imbalance in the actual data stream is reflected, it can lead to poor average precision during deep learning classification. Therefore, it was necessary to balance the dataset with nearly equal data over different classes. The high-resolution drone images have a spatial resolution of 4000 × 3000 pixels. The images were resized and scaled using bilinear interpolation [55] to make them compatible with the input size of the base models; in this work, it was set as 150 × 150. Using bilinear interpolation, the size of the images was reduced without affecting the characteristics of the actual high-resolution images and thus the features are preserved for the deep neural network classification. Then the images were boosted and augmented using 4 different transformations: rotation, flipping, shifting, and shear. The transformations were applied iteratively for different classes by changing the parameters (rotation range, flip direction, shift range and direction, shear range) of each transformation.

Given *F_n_* is the number of images in the class type *C_i, i_*_=[1,2,…,18]_ of the original dataset and *Y_n_* is the number of transformations applied to each image *X_k_*_∈_ *_Ci, i_*_=[1,2,…,18]_, the total number of images *D_n_* in the augmented dataset is based on Equations (1) and (2). The procedure will augment entries from the minority classes to match the quantity of the majority classes without overfitting the oversampled classes and ensure that no image is repeated. Thus, a total of 68,356 different images were generated from 2233 images.

(1) $$Y_{n}=\frac{\mathrm{Number}\;\mathrm{of}\;\mathrm{Iterations}}{\mathrm{Batch}\;\mathrm{Size}\;\mathrm{of}\;\mathrm{the}\;\mathrm{Images}}$$

*D_n_ = Y_n_ × F_n_* (2)

**Figure 5:**
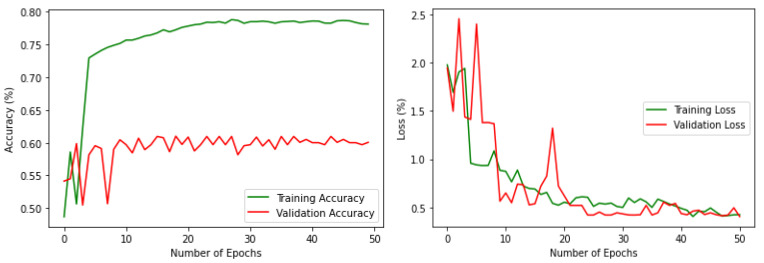
Accuracy and loss, respectively, for the processed data.

**Figure 6:**
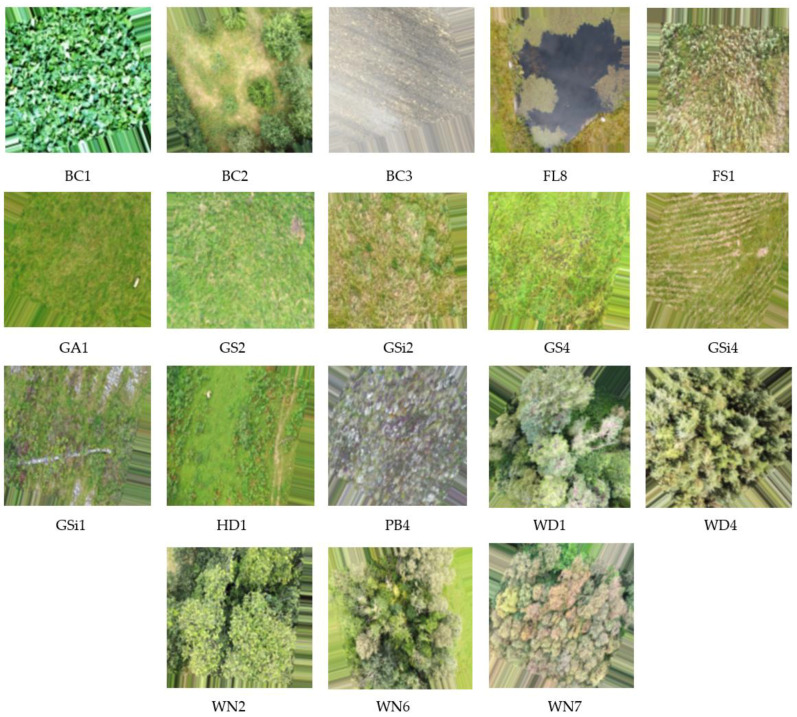
Sample images of 18 different habitats in the dataset (from Figure 2) following transformation using a combination of rotation, flipping, shifting, and shear.

For the processed dataset, preliminary training and validation were performed with the same VGG16 model to compare the performance with the imbalanced one. The results are shown in Figure 5 which demonstrates the improvement in both training and validation. It is noteworthy that the validation accuracy is increased to 60% by rectifying the data imbalance problem to a certain extent. Figure 6 shows one sample image from each of the habitats along with habitat type in the processed and augmented dataset.

### 2.3. Model Selection and Tuning

The multi-CNN approach proposed in this work is based on a set of pre-trained CNN models selected by hyperparameter tuning and customized using the transfer learning approach. There are many pre-trained models available for image classification such as VGG [56], Inception [57], Xception [58], Mobilenet [59], Resnet [60], DenseNet [61], SquezeNet [62], Shufflenet [63], and many others trained using Imagenet [64], which has more than a million natural images belonging to 1000 different classes. Out of these, 2 comparatively lightweight yet accurate-enough models for classification, VGG16 [56] and ResNet34 [60], were chosen along with MobilenetV2 [58], taking into account that the extended work has to be implemented in embedded boards and eventually in a mobile phone.

CNN models consisting of convolutional, pooling, and fully connected (FC) layers can be efficiently used for image classification [65]. The 2D convolutional layer extracts the local patterns of input features through several feature maps and kernels. This extracted feature vector is then compressed to low resolution by the pooling layer. Pooling helps to decrease the computational cost and over-fitting. Then, these features are given as input to the FC layers. In this work, since the habitat dataset is different compared to the Imagenet database, the final FC layers were removed, and a transfer learning approach was implemented, where pre-trained models are used as the starting point for training and subsequently re-modelled for the specific task. For the specified classification problem, a feed-forward DNN [66] with various layers and neurons was tried, and finally 3 hidden layers with 512, 256, 128, and 64 neurons respectively were chosen as the end layers of each of the model. A DNN model is suitable for processing nonlinear data, and the performance is better for classification problems. The newly added fully connected dense layers learn the linear and nonlinear relationship between the input features and target, whereas the weights of the convolutional layers of the pre-trained network are kept frozen. In order to prevent overfitting, a dropout layer of 0.2 is added in between the layers. Dropout [67] is a more effective and computationally inexpensive regularizer that prevents overfitting. All layers used rectified linear unit (ReLU), which is the most commonly used non-linear activation function [68]. The final classification layer used a SoftMax activation function [68] with 18 neurons corresponding to the number of classes. All the models are trained for a maximum of 50 epochs with a batch size of 32, where the number of steps in each epoch is fixed using Equation (3). Overtraining of the models during training is avoided by early stopping [69]. Early stopping is a method that allows specifying an arbitrarily large number of training epochs and ceasing training once the model performance stops improving on a holdout validation dataset.

(3) $$\mathrm{Number}\;\mathrm{of}\;\mathrm{Steps}\;\mathrm{in}\;\mathrm{each}\;\mathrm{epoch}=\frac{\mathrm{Number}\;\mathrm{of}\;\mathrm{Images}\;\mathrm{in}\;\mathrm{the}\;\mathrm{dataset}}{\mathrm{Batch}\;\mathrm{Size}\;\mathrm{of}\;\mathrm{the}\;\mathrm{Images}}$$

**Figure 7:**
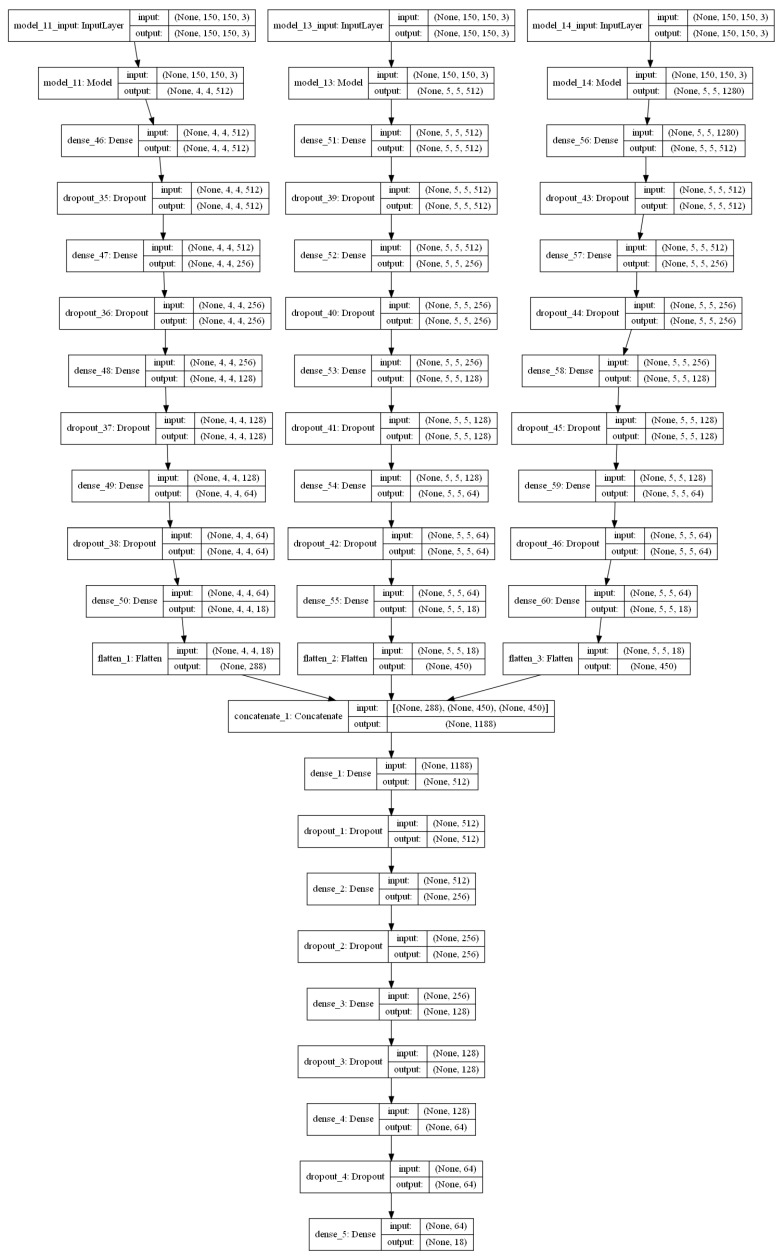
Stacked ensemble model.

Since the dimensions of the input images are scaled to 150 × 150 during the pre-processing step, the base models are also modified to make them compatible with the input size. Based on the architecture, the 3 base models with VGG16, ResNet34 and MobileNetV2 produced feature vectors of 4 × 4 × 18, 5 × 5 × 18, and 5 × 5 × 18, respectively, for every image. This is given as input to the final classification layer. The architecture of each model can be understood from Figure 7.

### 2.4. Hyperparameter Tuning

Each model was trained with different optimizers by varying the learning rates and the best-fit hyperparameters were chosen as the ones with the highest validation accuracy. Based on previous work in image classification, the four most suitable optimizers, namely, Adam, RMSProp, SGD, and Nadam were chosen for tuning each model [70,71,72]. The learning rate was varied from 10^−2^ to 10^−5^ to determine the best fit. Since the problem is image classification, categorical cross-entropy was taken as the loss function for training, due to the fact that one sample can be considered to belong to a specific category with probability 1, and other categories with probability 0. The results of the validation accuracy are given in Table 2. From the Table it can be seen that the Adam optimizer with a learning rate of 10^−4^ and 10^−3^ is the best-fit for the base models with VGG16 and ResNet34 respectively; for the base model with MobilenetV2, RmsProp with a learning rate of 10^−3^ is the best-fit.

### 2.5. Stacked Ensemble Model

Stacking the base models reduces the dispersion of the predictions and can make better predictions than any single contributing model [73]. Ideally, a model with low bias and low variance will provide better performance, although in practice it is very challenging and often difficult to achieve. Ensemble models provide a way to reduce the variance of the predictions, leading to improved predictive performance. Ensemble models can be implemented by averaging the individual models, where each model essentially contributes equally to the ensemble prediction, despite the performance of each model. Motivated by this, another approach is a weighted average ensemble, where the models which perform well are required to contribute more, while worse-performing models are required to contribute less. A further generalization of this approach is stacked ensemble which combines the predictions of the sub-models to generate new predictions.

The proposed stacked ensemble learning process consists of two-level stacking. In the first level, 3 base models are trained to obtain the initially predicted features. In the second level, a feed-forward DNN model is used as the meta-learner trained with the combination of predicted features of the first level to obtain the final classification result. The feed-forward DNN of the meta-learner is also designed with 3 hidden layers and a final classification layer similar to the base-learners. The optimizer and learning rate are tuned similar to the base-learners to derive the best fit by considering validation accuracy as reference. The number of nodes and all other parameters is set in the same way as that of base-learners. Based on the results presented in Table 2, Adam optimizer with a learning rate of 10^−4^ demonstrated better performance. The architecture of the stacked ensemble model is shown in Figure 7. Increasing the number of levels of the stacking will improve the performance of the model up to a certain limit which must be found experimentally, but it also increases the execution time. Also, since each level demands different datasets, for this particular work, the number of stacking levels is confined to 2 because of the limitations of the dataset mentioned in Section 2.2.

**Figure 8:**
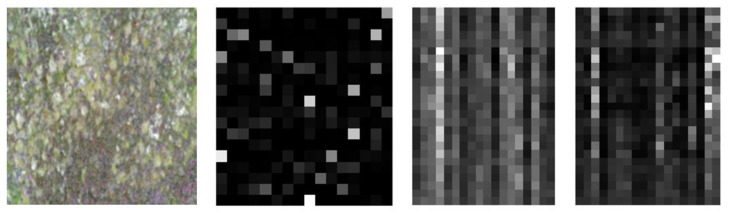
Sample Image from PB4 and its predicted features for base-learners with VGG16, Resnet34 and MobilenetV2 respectively.

The pre-processed data was split into training, validation, and test data with a ratio of 7:2:1 to ensure different datasets as input to different levels. While training the base-learners, the hidden layers were not flattened as the input is a 2D image, which has significant importance for its spatial coordinates corresponding to brightness, edges, and corners. The predicted features for a sample image are shown in Figure 8. The feature vectors of size 4 × 4 × 18 and 5 × 5 × 18 respectively (refer to the previous Section 2.3) are reshaped to 2D vectors of size (16,18) and (25,18), respectively, for visualization. The base models are trained with training data and the model is saved.

For training the meta-learner, validation data was fed to the already trained base models to obtain the preliminary predictions. Then these initial predictions were concatenated to get a single dataset which was then used to train the meta-learner. The weights of the meta-learner were optimized based on target classes. After training the base models and meta-learner, the test data was given for performance evaluation of the stacked ensemble model. The test data was first given to the base models to obtain the preliminary classification probabilities, which were then combined and fed to the meta learner to get final predictions.

## 3. Results

**Figure 9:**
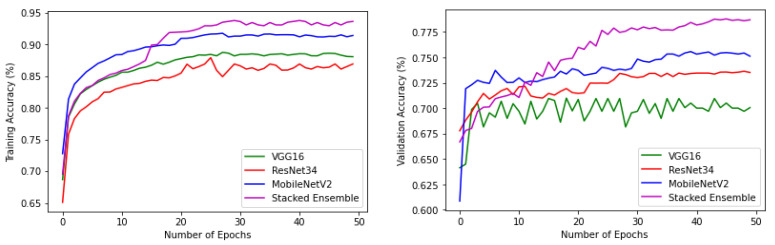
Training and validation accuracy of base-learners with VGG16, Resnet34, MobilenetV2, and Ensemble models, respectively.

**Figure 10:**
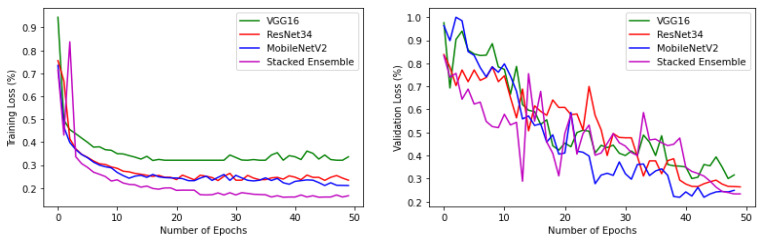
Training and validation loss of base-learners with VGG16, Resnet34, MobilenetV2, and Ensemble models, respectively.

The experiments were conducted using a core i7, GTX 1650 Graphics Processing Unit (GPU) with 16 GB RAM. The weights of each model were saved when it achieved maximum validation accuracy, preventing it from overfitting. The variation of accuracy and loss with respect to the number of epochs for training and validation are shown in Figure 9 and Figure 10. For all models, the training step was carried out to 50 epochs. It can be seen from the results that the highest accuracy and lowest loss for both training and validation were obtained for the stacked ensemble model. The accuracy was improved considerably for the meta-learner.

The performance metrics used in this study to evaluate the models are accuracy, precision, recall, *F1*-score, and area under the receiver operating characteristic curve (AUC). Precision is the percentage of samples classified in a class that were actually members of that class and recall is the percentage of samples of a class that were classified as that class by the model. *F1*-score, the summary of recall and precision, is the weighted average between these two values. Accuracy is the percentage of correct predictions across the whole dataset. They are defined using Equations (4)–(7).

(4) $$Precision=\frac{TP}{TP+FP}$$

(5) $$Recall=\frac{TP}{TP+FN}$$

(6) $$F1\;Score=2\times \frac{Precision\times Recall}{Precision+Recall}$$

(7) $$Accuracy=\frac{TN+TP}{TN+FP+TP+FN}$$

where *TP* is the number of true positives, *TN* is the number of true negatives, *FP* is the number of false positives, and *FN* is the number of false negatives. Values close to 1 indicate good performance. These values are calculated from the confusion matrix which describes the complete performance of the model.

**Figure 11:**
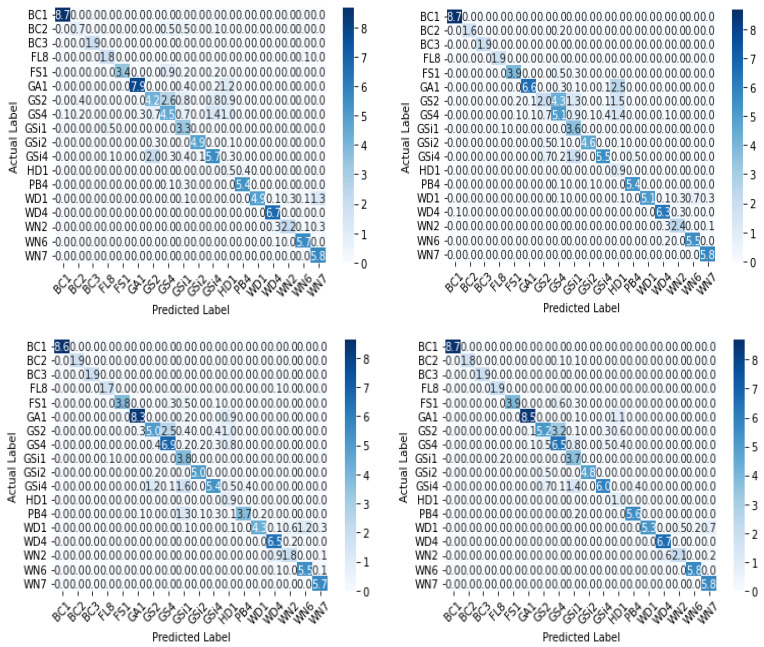
Confusion matrix (in %) of base-learners with VGG16, Resnet34, MobilenetV2, and Ensemble models, respectively.

The confusion matrix of the base-learners along with the ensemble model is shown in Figure 11. Table 3 describes class-wise accuracy values of the performance metrics. It is calculated by taking the average of values lying across the main diagonal of the confusion matrix, which is the ratio of total correct predictions to total predictions made. Table 4 depicts the comparison of results achieved using various base-learners in combination with the stacked ensemble model.

**Figure 12:**
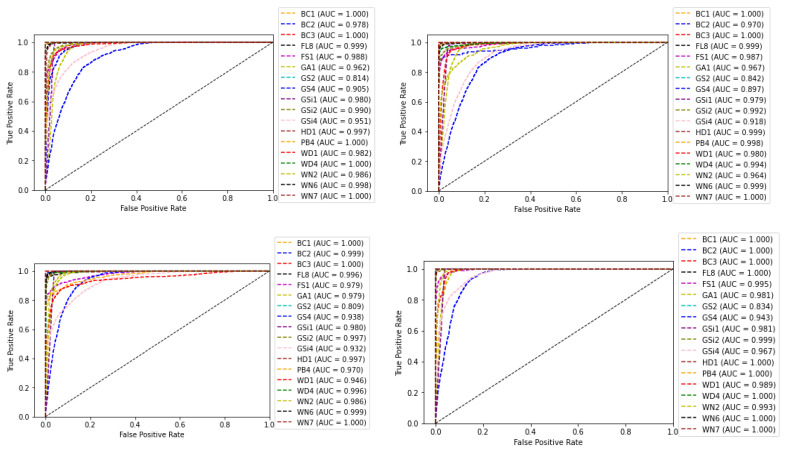
ROC curve and AUC values of base-learners with VGG16, Resnet34, MobilenetV2, and Ensemble models, respectively.

The receiver operating characteristic (ROC) curve, which is a plot between false positive rate (FPR) and true positive rate (TPR) corresponding to the four classifiers, is shown in Figure 12. The area under the ROC curve (AUC) for each class is also shown along with the plots. AUC measures the entire two-dimensional area underneath the ROC curve from (0,0) to (1,1). The value ranges from 0 to 1, representing the probability that the model ranks a random positive sample more highly than a random negative sample.

The number of parameters and execution time for 1 epoch along with classification accuracy are compared in Table 5 for each of the models. This will provide valuable insights for developing efficient edge algorithms in the future where these values will be a major concern in the model design. It can be seen that the base-learner with MobilenetV2 had the lowest execution time among all the models and the highest accuracy among the base models. The base-learner with Resnet34 had the highest execution time among all the models but its accuracy was even lower than the stacked ensemble one. The second highest execution time was for the stacked ensemble model which is obvious from the number of levels to train. However, when compared with its high accuracy the execution time is satisfactory.

Table 6 displays the results of major state-of-the-art methods published not long ago, along with the proposed best performing stacked ensemble model. Most of the existing methods deal with marine and land habitats and none of them addresses the problem of farmland habitat classification. The dataset, number of samples, and validation techniques used by the various state-of-the-art methods are different. Therefore, a fair comparison of the results is not possible. Though the habitat types are different, it is noteworthy that the proposed method has proven its effectiveness.

**Figure 13:**
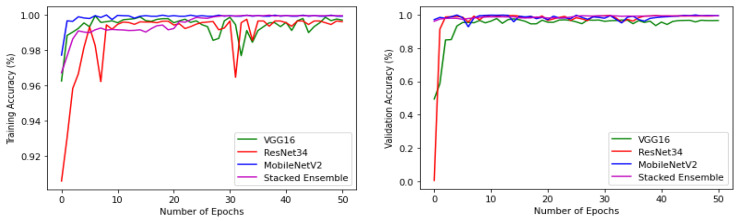
Training and validation accuracy of base-learners with VGG16, Resnet34, MobilenetV2, and Ensemble models, respectively, for the reduced dataset.

**Figure 14:**
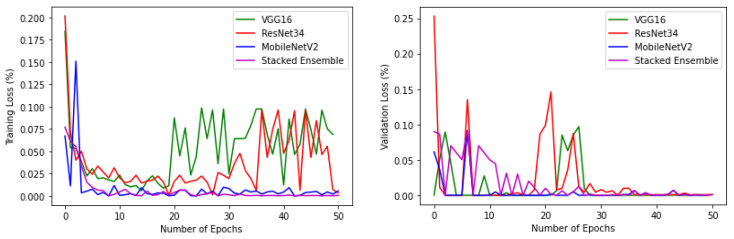
Training and validation loss of base-learners with VGG16, Resnet34, MobilenetV2, and Ensemble models, respectively, for the reduced dataset.

**Figure 15:**
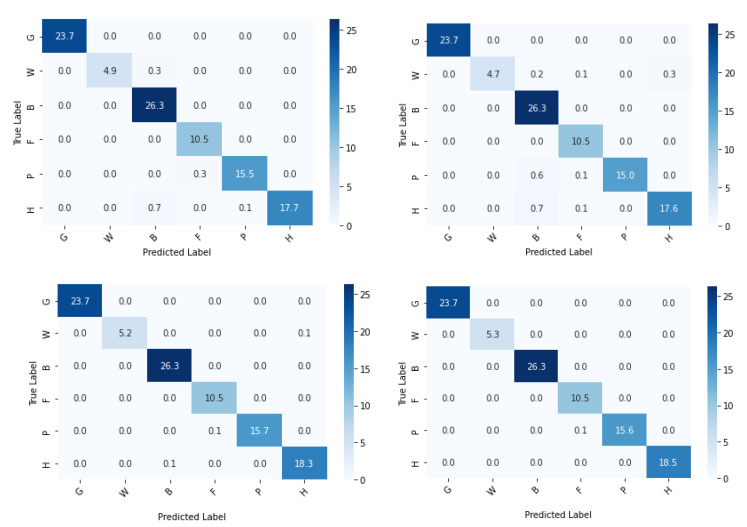
Confusion matrix (%) of base-learners with VGG16, Resnet34, MobilenetV2, and Ensemble models, respectively, for the reduced dataset.

**Figure 16:**
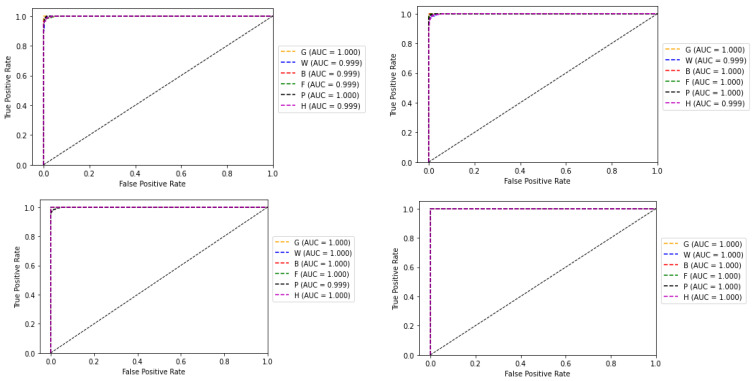
ROC curve and AUC values of base-learners with VGG16, Resnet34, MobilenetV2, and Ensemble models, respectively, for the reduced dataset.

**Figure 17:**
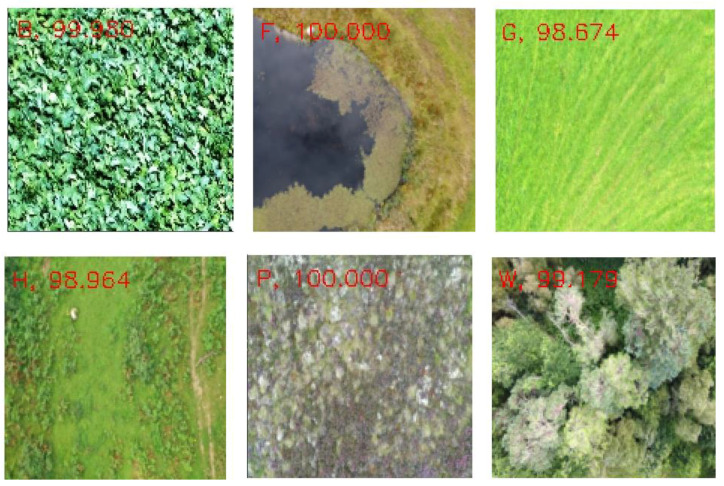
Classification accuracy (%) of sample images.

Another experiment was conducted, by choosing a single subclass from each category and applying the same algorithms and models described in Section 2 to the reduced dataset to classify the samples into six broader classes, as described in Table 1. The variation of accuracy and loss with respect to the number of epochs for training and validation are shown in Figure 13 and Figure 14 respectively. The confusion matrix of the base-learners along with the ensemble model is shown in Figure 15. It can be seen that all the models show very good results. The AUC approached 1 with nearly 0 misclassifications for the stacked ensemble model (Figure 16). Figure 17 shows some of the sample classification results along with the classification accuracy for the second experiment in which the habitats are classified into each of the broad categories.

## 4. Discussion

From the results, it was verified that the performance of the stacked ensemble model outperforms the base-learners. The overall prediction accuracy achieved by the stacked ensemble model was 88.44%. Some classes were predicted well, whereas the prediction of other classes was not so good, which ultimately affects the overall performance of the model (Table 3). For any deep learning model, during the training or the validation phase, the model can overfit as it tries to represent as much as possible the underlying characteristics of training data. This process deeply downgrades the model’s ability to perform accurate predictions on new data. Although techniques to prevent the model overfitting (such as using three different datasets at the three levels of training, data augmentation and transformation, dropout, and early stopping), deep learning models produce better results only when given a vast number of samples in the training phase. Therefore, the main reason for this is the imbalance of the self-collected dataset that was used for the work, which is clear from Figure 3. The other reason is that the method needs to classify many subclasses that belong to different broad categories which are often confusing. However, this could be overcome by the collection and addition of more images from under-represented classes, and further development of this preliminary approach.

As seen from Table 1, the broader classes have many sub-categories within them. Sub-classes belonging to the same broad category have very similar features for which the models find it difficult to establish distinguishable patterns between them. Heterogeneity of samples within the same class is another reason for misclassification. This is because of the dynamic nature of the farmland habitats which makes the acquisition system capture the same habitat types in slightly varying formats at different time intervals. This was overcome to a certain extent by collecting the data in the same season and almost at the same time throughout the data collection period. In addition, certain samples that showed high variance compared to other samples in the same class were manually removed. However, variations within the habitats still exist.

A lot of misclassifications can be seen in the middle blocks of the confusion matrix. The reason is that the middle row constitutes G: grassland and marsh, comprising six subclasses, which is the most in this experiment, all of which have nearly indistinguishable features. In this category, GSi1 had the highest prediction accuracy as it is dry calcareous and neutral grassland with rocky features, not seen in any other habitat types. Misclassifications can be seen in the bottom blocks of the confusion matrix also which constitutes W: woodland and scrub, which has the second-highest number of sub-classes—five, in which WD1 and WN2 have similar features contributing to prediction errors. Though the habitat mapping problem is a challenging task, from Table 6, it can be understood that the proposed method performed well.

Based on the results provided in Table 5, if the execution is done on a GPU machine, the stacked ensemble model will always be a good choice considering the high accuracy. However, when there is a trade-off between execution time and accuracy, especially for resource-constrained edge devices, MobilenetV2 may be a good choice. The classification accuracy of the stacked ensemble model is around 4% higher than the base-learner with MobilenetV2 whereas its execution time is approximately 6 min lower. However, more experiments need to be performed for better understanding in this regard and other models need to be explored.

In the second experiment when the number of classes was reduced, accuracy approached approximately 100% with no misclassifications. This can be verified from the associated results shown from Figure 13, Figure 14, Figure 15, Figure 16 and Figure 17. From this experiment, it is clear that the imbalance of the data, similar features between subclasses, and varying features within the same classes decrease the prediction accuracy of the habitat mapping process.

## 5. Conclusions

We investigated an approach for farmland habitat classification which is an important contribution to improved monitoring and preservation of ecosystems. High-resolution self-collected drone images were used collected over 18 different habitat types that were grouped into six broad categories. A stacked ensemble model was proposed which reduced the bias and variance and thus improved the prediction accuracy. The average accuracy over all the classes reported was 88.31% using this approach. Although an effective farmland habitat classification technique is proposed, there is room for further work. The data set used in this work is an imbalanced one with only 2233 samples across a comparatively large number of classes. More samples need to be collected that should be balanced across all possible habitat types to improve the accuracy of the deep learning technique. As an extension of this work, we aim to collect more samples and label them to make the dataset publicly available so that the entire research community can benefit. Other state-of-the-art pre-trained CNN architectures could be tried and various combinations of pre-trained CNNs could be explored in the future to understand the best possible model that will adapt the minute variations of the features among different habitats. In this work, tuning was conducted only for identifying the best optimizer and learning rate. Extensive hyper-parameter tuning could be conducted to develop the best fit model for particular applications. Different mechanisms to develop resource-efficient, deep learning models for mobile edge devices and other IoT devices used in smart agriculture have to be investigated. Based on the requirements of the execution environment (e.g., mobile phone or UAV capturing farmland images), a suitable compressed deep learning model can be generated.

## Figures and Tables

**Table 1:** Habitat Types of the Image Samples.

Class	Sub-Class	Species
G: Grassland and Marsh	GA1	Improved agricultural grassland
	GSi1	Dry calcareous and neutral grassland
	GS4/GSi4	Wet grassland
	GS2/GSi2	Dry meadows and grassy verges
W: Woodland and Scrub	WN2	Oak-ash-hazel woodland
	WN6	Wet willow-alder-ash woodland
	WN7	Bog woodland
	WD1	(Mixed) Broadleaved woodland
	WD4	Conifer plantation
B: Cultivated and built land	BC1	Arable Crops
	BC2	Horticulture Land
	BC3	Tilled Land
F: Freshwater	FS1	Reeds and large sedge swamps
	FL8	Other artificial lakes
P: Peatlands	PB4	Cutover Bog
H: Heath and dense bracken	HD1	Dense Bracken

**Table 2:** Hyperparameter Tuning Results. Best results achieved for each classifier is shown in bold.

Model	Optimizer	Validation Accuracy for Different Learning Rates			
		**10^−2^**	**10^−3^**	**10^−4^**	**10^−5^**
Base model with VGG16	**ADAM**	65.12	68.51	**70.05**	66.32
	RmsProp	62.51	64.62	63.29	61.52
	SGD	44.07	40.54	45.33	42.06
	NADAM	64.89	66.11	65.24	62.61
Base model with ResNet34	**ADAM**	69.06	**73.89**	72.32	71.54
	RmsProp	70.16	72.51	73.01	71.11
	SGD	56.55	58.34	58.22	57.51
	NADAM	61.21	73.51	61.37	60.29
Base model with MobileNetV2	ADAM	65.55	66.02	64.66	62.54
	**RmsProp**	69.18	**75.12**	73.51	72.66
	SGD	49.32	53.51	54.34	52.21
	NADAM	57.51	65.07	67.98	65.55
Stacked Ensemble	**ADAM**	75.54	77.02	**78.70**	77.37
	RmsProp	76.57	78.23	79.08	75.05
	SGD	63.39	64.21	64.11	65.63
	NADAM	69.54	69.29	65.95	63.49

**Table 3:** Class-wise accuracy of base-learners with VGG16, Resnet34, MobilenetV2, and Ensemble models.

Class	Model			
	VGG16	ResNet34	MobileNetV2	Ensemble
BC1	1	1	1	1
BC2	0.371	0.85	0.968	0.925
BC3	1	1	0.993	1
FL8	0.946	0.981	0.887	0.993
FS1	0.715	0.81	0.798	0.818
GA1	0.819	0.688	0.871	0.881
GS2	0.433	0.208	0.521	0.543
GS4	0.511	0.576	0.779	0.727
GSi1	0.856	0.948	0.979	0.948
GSi2	0.923	0.875	0.945	0.912
GSi4	0.637	0.623	0.575	0.699
HD1	0.531	0.962	0.968	0.993
PB4	0.934	0.931	0.646	0.962
WD1	0.724	0.755	0.647	0.778
WD4	1	0.933	0.965	0.993
WN2	0.756	0.841	0.641	0.710
WN6	0.987	0.956	0.954	0.993
WN7	1	0.998	1	1

**Table 4:** Comparison of results achieved for various base-learners in combination with proposed classifiers. Best results achieved using the Stacked Ensemble model is indicated in bold.

Model	Performance Metrics			
	Precision	Recall	F–Measure	Accuracy
VGG16	0.8019	0.7647	0.7584	0.7861
Resnet34	0.8489	0.8451	0.8273	0.8300
MobilenetV2	0.8443	0.8611	0.82	0.8414
**Stacked Ensemble**	**0.8944**	**0.9016**	**0.8842**	**0.8844**

**Table 5:** Comparison of execution time and accuracy for various models. Best results are indicated in bold.

Model	Number of Parameters		Execution Time in Minutes/Epoch		Accuracy
	Total	Non-Trainable	Trainable		
VGG16	18,662,906	14,714,688	3,948,216	3.41	0.7861
Resnet34	27,684,411	21,302,473	6,381,938	5.57	0.8300
MobilenetV2	18,086,322	12,257,984	5,828,338	**1.04**	0.8414
Ensemble	40,759,762	39,977,279	782,483	5.50	**0.8844**

**Table 6:** Comparison of results with other recent state-of-the-art methods.

Study	Habitat Type	Dataset	Classes	Classifier	Model	Accuracy
A.Diegues et al., 2018 [45]	marine	Self-collected	2	CNN	VGG16	85.10
T.Liu et al., 2018 [37]	wetland	Self-Collected	7	CNN	-	76.90
A. Gómez-Ríos et al., 2019 [43]	marine	EILAT & RSMAS	8, 14	CNN	ResNet50 & ResNet151	98.90 ^1^
M. Yasir et al., 2020 [38]	marine	MLC	9	MLP	DenseNet169	87.40
This study *	**farmland**	**Self-collected**	**18**	**CNN**	**Stacked Ensemble**	**88.44**

^1^ Combined accuracy for all datasets; * Results achieved using proposed method is indicated in bold.

## Data Availability

The dataset will be made available upon request.
